# Interfacing Neural Network Components and Nucleic Acids

**DOI:** 10.3389/fbioe.2017.00053

**Published:** 2017-12-04

**Authors:** Thomas Lissek

**Affiliations:** ^1^Department of Neurobiology, Interdisciplinary Center for Neurosciences, Heidelberg University, Heidelberg, Germany

**Keywords:** DNA, brain, neural networks, computation, nucleic acid, genome, information, medicine

## Abstract

Translating neural activity into nucleic acid modifications in a controlled manner harbors unique advantages for basic neurobiology and bioengineering. It would allow for a new generation of biological computers that store output in ultra-compact and long-lived DNA and enable the investigation of animal nervous systems at unprecedented scales. Furthermore, by exploiting the ability of DNA to precisely influence neuronal activity and structure, it could be possible to more effectively create cellular therapy approaches for psychiatric diseases that are currently difficult to treat.

## Introduction

In the vertebrate brain, two of nature’s most versatile and powerful information processing systems meet—neural membranes and nucleic acids. By interfacing these two systems, evolution has made possible the astounding feats of complex animal behavior and higher cognition. In the last three decades, neurogenomics has elucidated the many ways in which neural membranes and nucleic acids can communicate with and influence each other in the course of nervous system development, neuronal survival, circuit function, and synaptic plasticity (Flavell and Greenberg, 2008; Hagenston and Bading, 2011). It also implicates a vast potential for biotechnological and biomedical applications and here I will first briefly review the many ways in which these two systems interface with each other under natural conditions to later explore their potential for synthetizing useful tools in data processing and medicine. I propose applications in the form of neuron-culture based computers that write and store output as long-lived and ultra-compact DNA and evaluate the potential of custom-writable neuron templates for correcting pathologic brain circuits.

## Basic Principles of Information Processing in Neural Circuits and in Nucleic Acids

**Figure 1:**
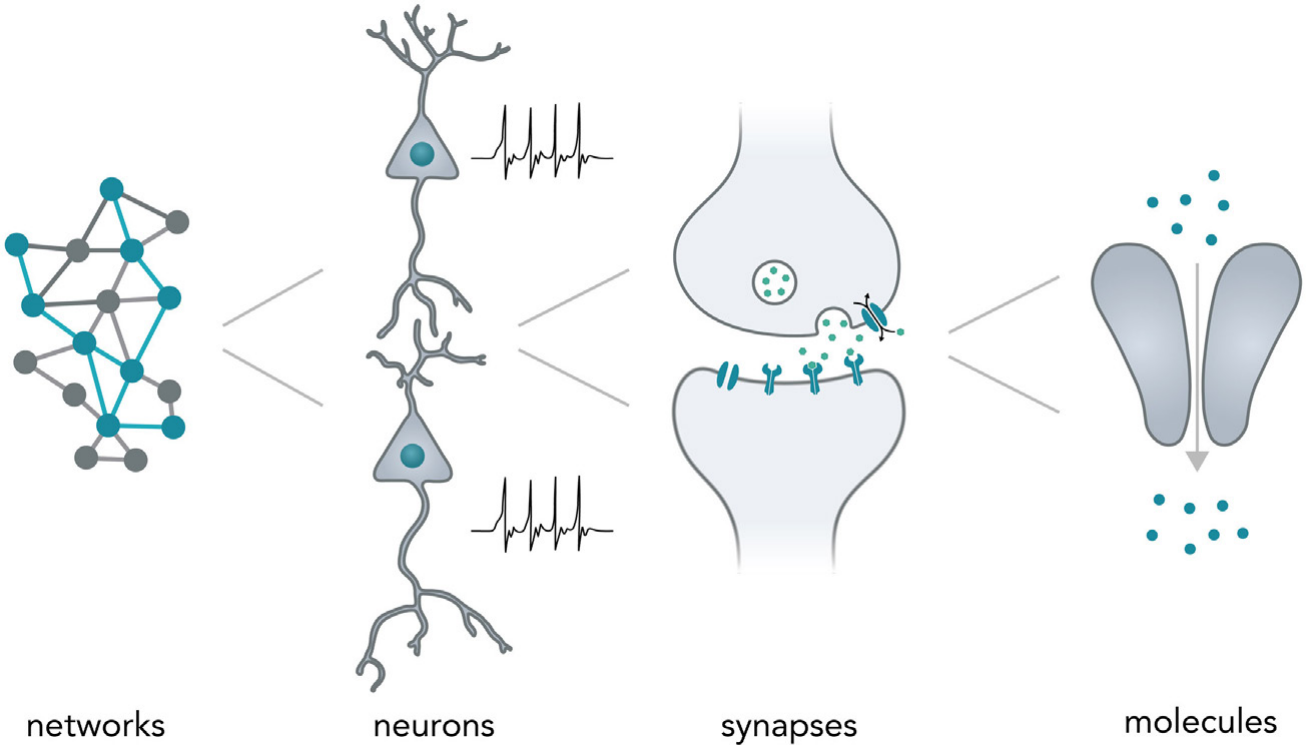
Neural computation. Shown are the different levels of information processing in the brain. At the highest level, coactivity of neurons in a network ensemble binds together vast amounts of information and is thought to incorporate several perceptional features (i.e., sound and vision) into one coherent mental construct. On the single neuron level, cells are able to integrate thousands of inputs in a non-linear fashion and encode information as changes in their membrane potential. Synapses between neurons are able to translate electrical membrane potential changes into chemical signals and realize the communication between neurons. Lastly, even individual neuronal molecules are able to perform computations, i.e., for *N*-Methyl-d-aspartate receptors: let calcium pass if and only if (a) glutamate is bound and (b) the membrane is depolarized.

Neurons typically form complex networks in order to process stimuli from the environment and influence an organisms behavior accordingly. Neural computation relies on processes on various levels—from network-wide ensemble activity over individual neuronal firing patterns and synaptic transmission to the activity of individual molecules (Figure 1). Data is processed on the millisecond timescale but can be stored over several decades in the form of long-term memory (Kandel, 2013). In the human brain, neural networks are so tightly packed that it allows around 85 billion neurons (Herculano-Houzel, 2009) and around 100 trillion synapses (Pakkenberg et al., 2003) to fit in a volume of 1.2 dm^3^ (Leonard et al., 2008) while weighing only 1.5 kg (Herculano-Houzel, 2009). Considering the vast computational power of even a single neuron (Koch and Segev, 2000) and the fact that the energy consumption of the whole human brain [20–25 W as based on metabolic activity reported in a previous study (Mink et al., 1981)] is far lower than for most modern table-top computers (~100 W), it is evident that biological neural networks hold vast potential for dense and energy-efficient information processing and storage.

**Figure 2:**
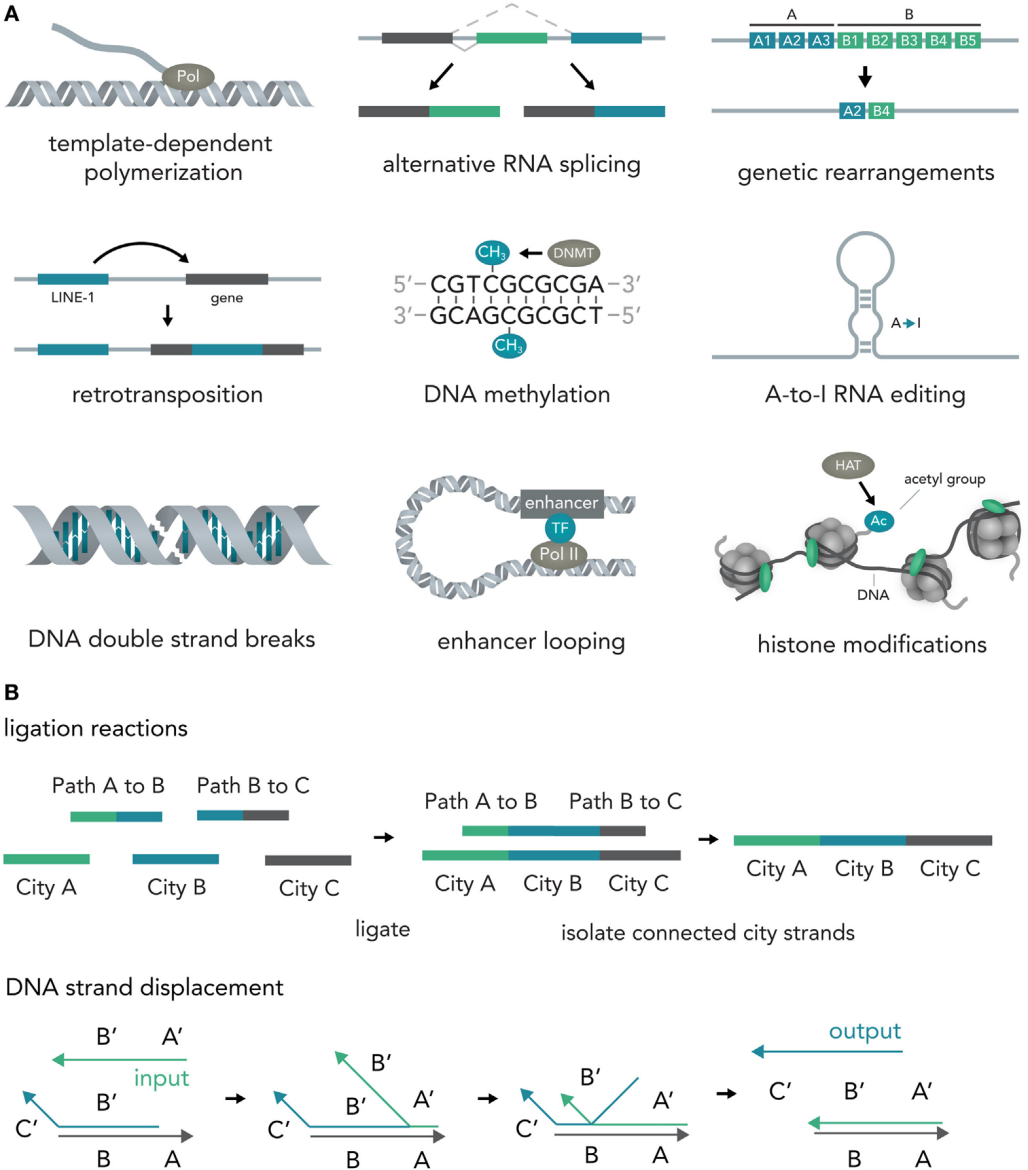
Writing and storing data in nucleic acids. **(A)** Natural computation mechanisms. In eukaryotic cells, information processing and storage in nucleic acids are realized in various ways. Template-dependent polymerization synthesizes new nucleic acid strands (i.e., RNA transcription or DNA duplication). Alternative RNA splicing generates different versions of a final transcript from a common precursor molecule. Genetic rearrangements lead to a final specific DNA sequence from initial precursor sequences (i.e., VDJ-recombination in B-cells). In retrotransposition, a specific genetic element (i.e., LINE-1) is duplicated and inserted into a target sequence. In the course of DNA methylation, cytosine residues become methylated by DNA methyl transferases (i.e., DNMT3A). A-to-I-RNA editing leads to transformation of an adenosine residue to an inosine and subsequently impacts the transcript function (i.e., miRNA targeting or mRNA translation). DNA double strand breaks (DSBs) can be induced at specific locations and can influence gene transcription. Enhancer looping refers to structural DNA changes in which sequences that are separated by long DNA segments are brought close together by looping of the DNA molecule (i.e., enhancers bringing transcription factors into close vicinity of the RNA polymerase complex). Histone modifications [i.e., acetylation via histone acetyl transferases (HATs)] allow the tuning of DNA accessibility and thus transcription. **(B)** Artificial nucleic acid computations. DNA ligation reactions are able to approximate solutions to various mathematical problems (i.e., the NP-complete Hamiltonian path problem). Different data points (here cities) and operations (here linkage of data points via complementary binding of linkers to half a city sequence) are represented by specific DNA sequences that are then ligated, isolated, and analyzed. Another method, DNA strand displacement cascades, makes use of predictable hybridization kinetics to transform inputs to outputs. These outputs can serve as inputs for downstream reactions and, thus, create complex computational networks.

In nucleic acids such as DNA and RNA, information is encoded in the specific sequence of bases—Adenine (A), Thymine (T) for DNA or Uracil (U) for RNA, Cytosine (C), and Guanine (G) (Alberts, 2015). Previous work has outlined the vast abilities of natural nucleic acid segments and epigenetic mechanisms to write and store information (Shapiro, 2006). In eukaryotic cells, these include template-dependent polymerase reactions such as RNA transcription (Hahn, 2004) and DNA replication (Masai et al., 2010), alternative RNA splicing (Matlin et al., 2005), DNA sequence rearrangements (Bassing et al., 2002), covalent modifications such as methylation of cytosine in CpG islands (Bird, 2002), A-to-I RNA editing (Nishikura, 2016), double-strand breaks (DSBs) (van Gent et al., 2001), duplication, jumping of and insertion of complete sequences such as L1 retrotransposons (Ostertag and Kazazian, 2001), structural changes such as looping of enhancers to other sequences several thousand bases away (Marsman and Horsfield, 2012; Mora et al., 2016), and histone modifications that influence DNA access such as methylation or acetylation (Bannister and Kouzarides, 2011) (Figure 2A). Several reports have also explored the use of DNA computations in non-natural contexts (usually performed in test tubes) and found that oligonucleotide-based ligation reactions might be useful in tackling NP-complete problems such as the Hamiltonian path problem (Adleman, 1994) and that strand displacement cascades are able to reliably distinguish between different four-bit patterns (Qian et al., 2011) (Figure 2B). In mammalian cells, complete nucleotide sequences are synthesized on the millisecond timescale (Maiuri et al., 2011) but can persist over millennia with little to no degradation (Paabo et al., 2004) [i.e., 80% of the wooly mammoth genome has been sequenced even though specimens were preserved for 4,000 years under non-laboratory conditions (Miller et al., 2008)]. The amount of information that can be stored in DNA per volume is extremely high with current experimental evidence for 5.5 × 10^15^ bits per mm^3^ (Church et al., 2012) and thus far surpasses all currently established technologies. Recent progress has allowed increasingly rapid and cheap *de novo* synthesis of long DNA fragments (Kosuri and Church, 2014) and harnessing the ability of natural systems to efficiently modify DNA [i.e., sequence or structural changes via Cas9 (Mali et al., 2013)] will help biotechnology and medicine to leverage the vast potential of nucleic acids to store and compute information.

## Natural Interfaces Between Neural Network Components and Nucleic Acids

### From Neural Network Components to Genes

With the astounding capabilities that neuronal networks and nucleic acids each have on their own, their capacity for information processing multiplies when they work in concert. The neuron, for instance, is capable of translating an outside signal into wide-spread changes in nucleic acid content, structure, and function. It receives inputs via neurotransmitters that are then translated into intracellular signals and relayed to the nucleus *via* diverse mechanisms such as Ca^2+^ fluxes (Hagenston and Bading, 2011) and kinase cascades (Flavell and Greenberg, 2008) (i.e., MAPK and CaMK pathways).

**Figure 3:**
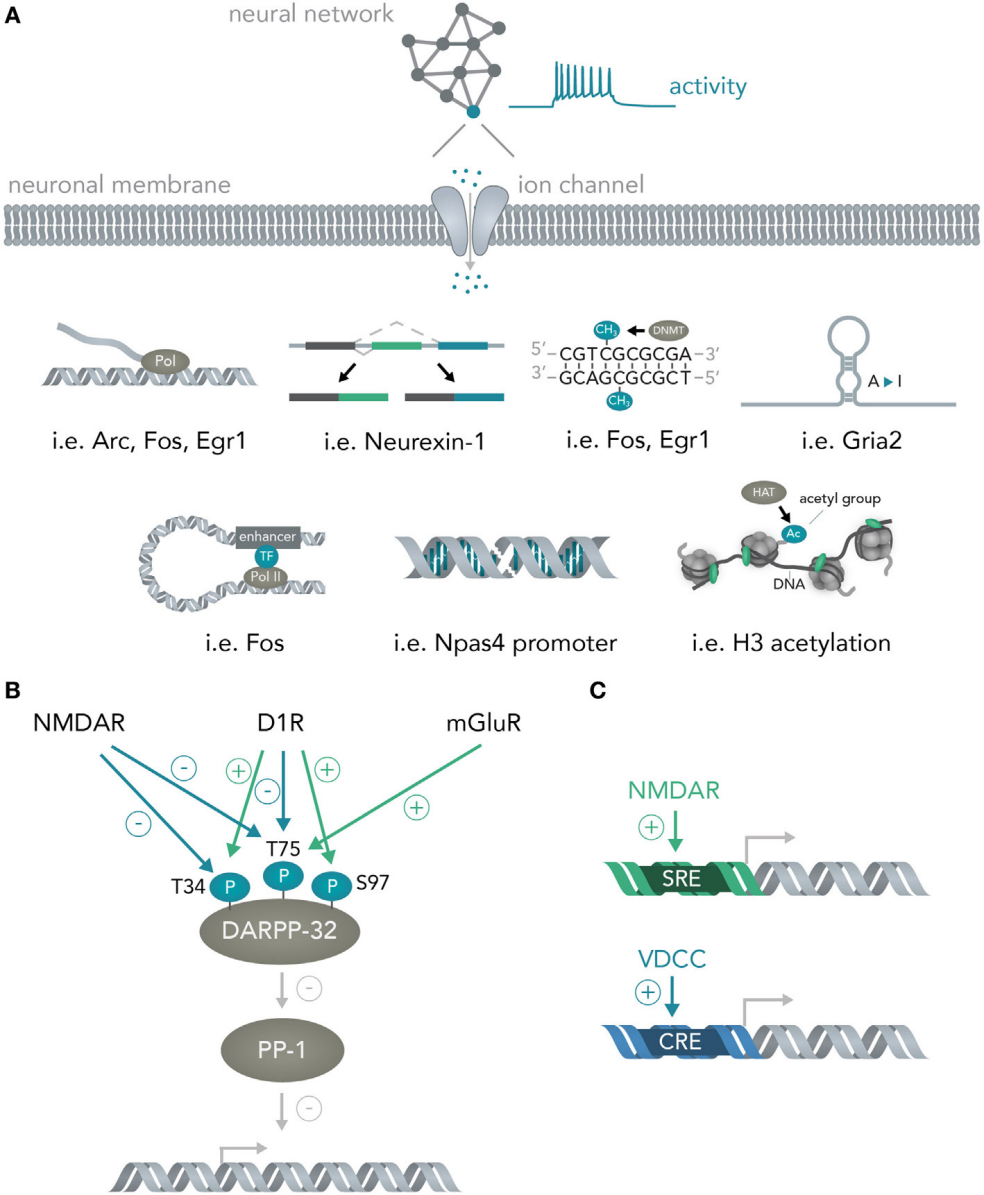
From neural networks to genes. **(A)** Translating neuronal activity into nucleic acid modifications. Network activity influences individual neuronal spiking which translates to opening of activity-dependent ion-channels. Subsequently, several nucleic acid computations are performed. Example genes are given for the mechanisms detailed in Figure 2A and include transcription of the genes Arc, Fos, Egr1, alternative splicing of Neurexin-1, DNA methylation of Fos and Egr1, A-to-I editing of Gria2 mRNA, enhancer looping to the Fos core promoter, DSBs in the Npas4 promoter and widespread H3 acetylation in the neuronal genome. **(B)** Integration of multiple neural network inputs by one neuronal molecule determines transcriptional activity. Activation of D1 receptors leads to phosphorylation of DARPP-32 at Threonine-(T)-34 and dephosphorylation at T75 whereas stimulation of *N*-Methyl-d-aspartate (NMDA) receptors leads to dephosphorylation of both these residues. Only activation of metabotropic glutamate receptors is able to induce phosphorylation of T75. Together with phosphorylation of Serine(S)-97 by D1 receptors, the phosphorylation state of these residues determine DARPP-32 interactions with its primary target protein-phosphatase 1 (PP-1) and, thus, regulate transcriptional activity. **(C)** Activation of different receptors leads to induction of distinct promoter elements. Ca^2+^ influx through NMDA receptors leads to induction of the serum response element (SRE) while not, or comparably less, inducing the cAMP responsive element (CRE). Ca^2+^ influx through voltage-dependent calcium channels (VDCCs) in turn leads to induction of the CRE in addition to the SRE. This allows inference of the channel activation (i.e. induction of only the SRE but not the CRE means NMDA receptors were activated, while additional CRE activation means VDCCs were activated).

As soon as the signal arrives in the nucleus, loops form from enhancers to promoters (Gray et al., 2015), histones are methylated (Malik et al., 2014), transcription of messenger RNAs for many immediate early genes is initiated by a diverse set of transcription factors (Hagenston and Bading, 2011) (i.e., CREB or SRF) and previously transcribed RNAs are alternatively spliced [i.e., Neurexin-1 (Iijima et al., 2011)] and modified [A-to-I editing of mRNAs for several immediate early genes (Sanjana et al., 2012)] (Figure 3A). Apart from activity-dependent transcription, recent reports have highlighted the role of synaptic activity induced changes in DNA methylation by the *de novo* DNA methyltransferase Dnmt3a (Oliveira et al., 2012; Day et al., 2013) and a study has found evidence for activity-induced DNA DSBs in which synaptic stimulation leads to DSB formation in the promoters of several immediate early genes and thereby facilitates their induction (Madabhushi et al., 2015). Every one of the above mechanisms could theoretically be used to convert the spiking activity of a neuron into sequence or structural information in a nucleic acid and, hence, allows bridging these two systems. In section 3, the possibilities and advantages of doing so will be discussed.

Importantly, the transcriptional cascades recruited and the specific genes transcribed often depend on the details of which circuits were activated and in what fashion. One example is the physiology of striatal medium spiny neurons. If D1 dopamine receptors are activated, the protein DARPP-32 (which is an inhibitor of protein phosphatase 1, and thus, able to regulate transcription of downstream genes) is specifically phosphorylated at Threonine-34 (Nishi et al., 1997; Svenningsson et al., 2004) and dephosphorylated at Threonine-75 (Svenningsson et al., 2004) (Figure 3B). Opening of *N*-Methyl-d-Aspartate (NMDA) receptors with subsequent activation of calcineurin dephosphorylates DARPP-32 at Thr-34 and Thr-75 (Svenningsson et al., 2004), whereas stimulation of metabotropic glutamate receptors leads to phosphorylation of Thr-75 (Svenningsson et al., 2004). Dephosphorylation at Ser-97 regulating nuclear localization of DARPP-32 together with the aforementioned phosphorylation at Thr-34 leads to inhibition of protein phosphatase 1 and allows this molecule to regulate transcription and neural network plasticity (Stipanovich et al., 2008; Graff et al., 2010). Thus, already one protein in neuronal cells can integrate several circuit-specific inputs and translate them into widespread changes in RNA content (Fernandez et al., 2006). As another example, calcium signaling initiated by two distinct channels, NMDA receptors and voltage-dependent calcium channels (VDCCs), induces discrete transcriptional elements (Bading et al., 1993); NMDA receptors induce transcription predominantly *via* the serum response element (SRE), while VDCCs, in addition to the SRE, seem to induce the Ca^2+^- and cAMP-responsive element (CRE). Determining the exact parameters that lead to activation of NMDARs versus VDCCs together with isolating the genetic elements and placing them into appropriate contexts will lead to opportunities for creating synthetic genetic modules that are able to predictably connect circuit activity to transcriptional logic operations (Figure 3C and Section 3). Furthermore, a study has shown that different elements within a c-fos enhancer (Joo et al., 2016) react to different neuronal stimuli and thus allow discrimination of distinct synaptic inputs (i.e., glutamate versus BDNF) at the genome level.

### From Genes to Networks

**Figure 4:**
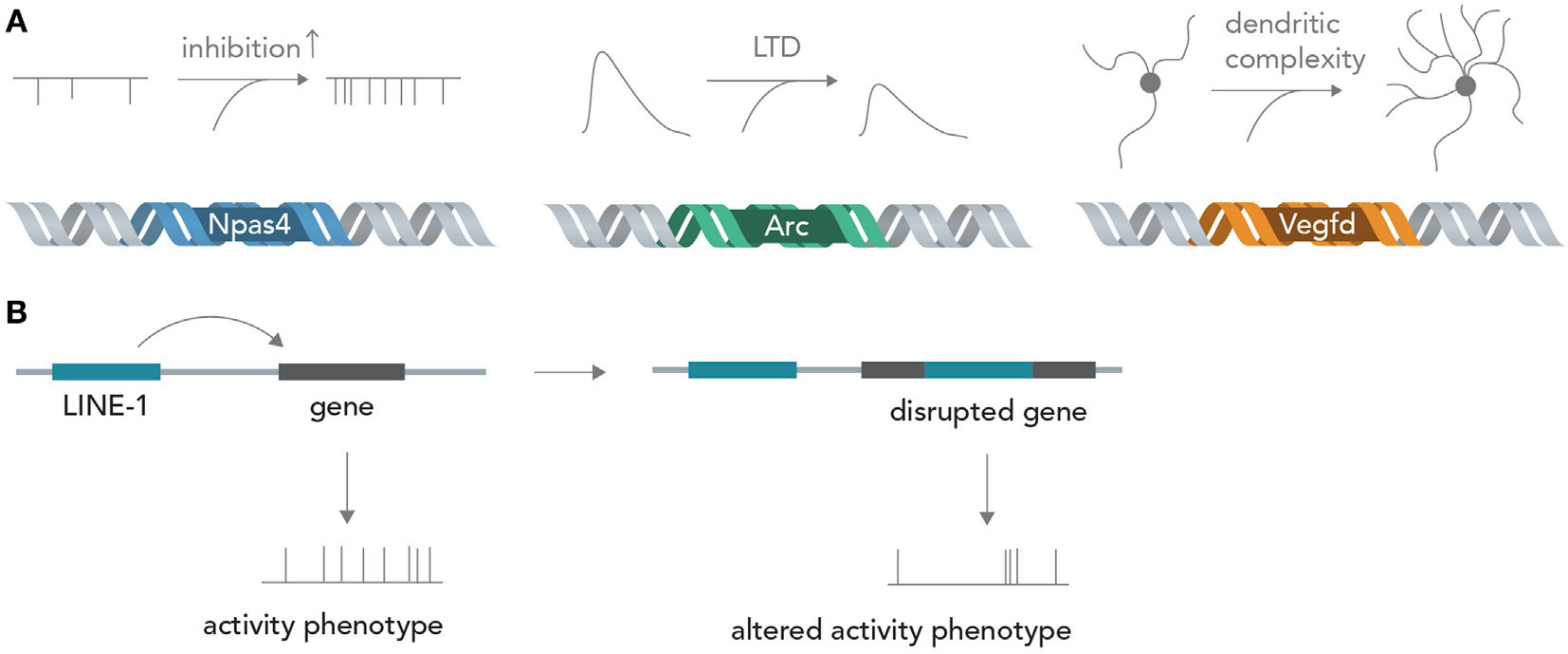
From genes to neural networks. **(A)** Genes affect neural function and structure. The activity-dependent transcription factor Npas4 leads, in excitatory neurons, to an increase in inhibitory inputs as measured by miniature inhibitory post-synaptic currents (mIPSCs), whereas the protein product of Arc/Agr3.1 leads to a reduction of synaptically evoked excitatory currents through long-term depression (LTD). Vegfd is able to regulate the dendritic structure of neurons and thereby influences the information content that can be received. **(B)** Retrotransposition can alter neuronal genomes. By disrupting functionally important genes in a seemingly random manner, LINE-1 elements could alter neuronal activity phenotypes.

Genes encode about every structural and functional aspect of the brain (Boguski and Jones, 2004). During brain development, information in DNA directs circuit wiring, establishes the correct overall architecture of brain regions and determines cell fate by differentiation into one particular out of many possible cell types (Kandel, 2013). Once the circuit is established in adult life, activity-dependent transcription of genes can profoundly influence neuronal function by determining synapse formation, ion channel composition, dendritic architecture, and metabolic state, among others (Flavell and Greenberg, 2008; Hagenston and Bading, 2011) (Figure 4A). For example, the acutely induced transcription factor Npas4 is able to tune excitatory and inhibitory input formation (Spiegel et al., 2014) and the gene product of Arg3.1/Arc is able to acutely decrease excitatory synaptic transmission *via* long-term depression (LTD) (Bramham et al., 2008). The protein product of *Vegfd* is able to regulate overall neuronal morphology, thereby exerting a deep influence on neural information processing (Mauceri et al., 2011). Recent studies also implied that L1 retrotransposition, which is able to directly alter the DNA sequence, has important functions in postnatal brain development and in adult brain function (Singer et al., 2010; Richardson et al., 2014) (Figure 4B). These few examples out of many show that the sequence information in DNA can exert profound influences on neural networks, both acute (i.e., by temporary transcription and incorporation of certain ion channels) and chronic (i.e., by changing long-term dendritic architecture and synapse formation), which could be exquisitely exploited for synthetic biology applications in which neural networks perform human-desired computations (see below). Changing the activity of circuit-altering genes by targeted modulation with Cas9 (Mali et al., 2013) or light-sensitive transcription factors (Konermann et al., 2013) could enable real-time modification of computational rules inherent in the neural network, and thus allow fine tuning of the network operation to achieve specific goals. The nucleic acids for many genes can nowadays be cloned or synthesized in a straightforward manner and introduced into neurons by various methods such as viral delivery, transfection or electroporation, making feasible the rapid and efficient neurogenomic programing with standard laboratory equipment.

## Neurogenomic Computers

### Basic Principles

**Figure 5:**
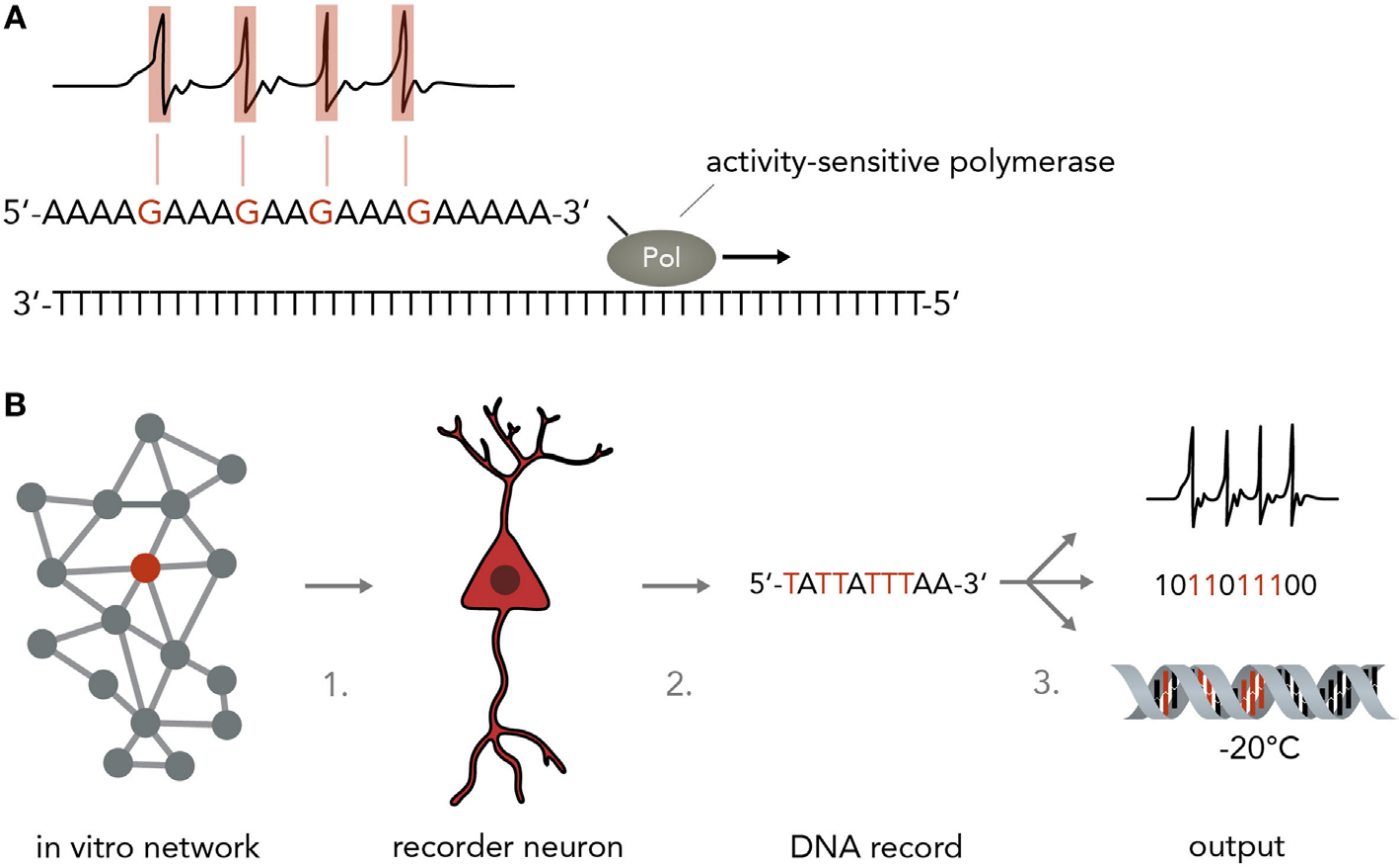
Neurogenomic computers. **(A)** Recording neural activity in nucleic acids. For instance, a stimulus-sensitive DNA polymerase as developed in a previous study (Zamft et al., 2012) (shown here is a simplified version to illustrate the principle more clearly) synthesizes from a template and incorporates a wrong base if the neuron is active (i.e., by making the polymerase error rate sensitive to intracellular Ca^2+^ concentrations). For simplicity, the template here is a poly-T string and synthesis errors are assumed to result in G incorporation instead of A. In reality, this might not be technically feasible and one might have to rely on statistical methods to infer the positions in which the polymerase performed with an increased error rate while synthesizing from a more complex template (i.e. containing A, T, C, and G). **(B)** Pipeline for implementing a neurogenomic recording device. In this biological computer, input is fed into the neuronal network via physical methods (i.e., electrical stimulation on a MEA or optogenetic activation). Either all neurons or one specific neuron (recorder neuron) express the activity-sensitive polymerase as mentioned in panel A. For information access, three steps would have to be performed. Step 1: a recorder neuron is harvested from the network after a computation is performed. To reconstitute network activity, it could be replaced with an immature neuron that then integrates into the circuit. Step 2: the DNA is isolated. Step 3: the information in the DNA could be sequenced and transformed into electronic bits, it could be transformed into neural activity or it could be frozen and stored and only sequenced when needed.

A simple model of a biological neuron-based computer is that of a dissociated neuron culture connected to a physical recording device such as a multi-electrode array (MEA) or a fluorescent microscope (although, strictly speaking, this is still a hybrid device). Reports have shown that such an *in vitro* network can be maintained over several months (Potter and DeMarse, 2001), stimulated in a highly controllable fashion (Wagenaar et al., 2004, 2005), used to navigate an airplane in flight-simulations (Demarse et al., 2001) and even steer a real-life robot through obstacle-ridden terrain (Novellino et al., 2007). Inputs into these networks are currently realized by electric or optogenetic stimulation, whereas the output is usually electric (in the form of patch-clamp or MEA recordings) or optical (i.e., by using fluorescent calcium or voltage indicators). One problem is that these recording techniques are usually limited to relatively few neurons at a time and necessitate extensive expertise, technical sophistication, and financial expenses. Recording around 200 neurons at single-cell resolution currently requires a MEA or a fluorescent microscope connected to a computer. It would be more favorable to use an output that allows simultaneous single-cell recordings of many neurons with inexpensive, fast and easy-to-implement methods. Herein lies a crucial advantage of using the natural interfaces between neural networks and nucleic acids described above. Over the past decades, research in nucleic acid analysis has brought forth extremely cost- and time-efficient methods to analyze DNA and RNA with high accuracy. These methods, such as DNA/RNA sequencing and qPCR analysis can be applied with little training and are relatively insensitive to external error sources. Translating the results of highly complicated and difficult-to-analyze neural network computations into nucleic acids will therefore be of immense benefit. In theory, any of the mechanisms discussed in Section “From Neural Networks to Genes” could be harnessed to convert neural activity into DNA/RNA changes, either by hijacking natural synapse-to-nucleus pathways such as the MAPK or CaMK cascades or by engineering artificial ones (O’Shaughnessy et al., 2011) that act orthogonally and thus do not interfere with the host cell physiology. An important point to consider is that the synaptic input in these cascades is usually amplified in a non-linear manner and integrated over time, which could be an advantage if the input signal is weak and extremely short-lived and needs to be enhanced or it could be a disadvantage if a more direct conversion of spiking activity and increased precision is desired. A line of work that might achieve a direct and undistorted conversion of synaptic inputs to nucleic acid modifications suggested and started to explore the use of molecular ticker-tapes in the form of DNA to record neural activity (Zamft et al., 2012; Marblestone et al., 2013) (Figure 5A). In one study, activity-sensitivity of the polymerase was realized by correlating the error-rate to cation concentrations so that increasing Mn^2+^ or Mg^2+^ would lead to a trace of wrongly incorporated bases (Zamft et al., 2012). Subsequent statistical analysis would then be used to infer the time spans during which the cation concentration was elevated and allow inference of the activity pattern of the neuron (Glaser et al., 2013). A possible variation would be to use a CRISPR-Cas9 based molecular memory device by implementing a self-targeting sgt-RNA system and storing the data as DNA mutation frequencies, as done in a previous study in mammalian cells (Perli et al., 2016). A recent report has demonstrated the use of converting the process of immunological CRISPR spacer acquisition in bacteria into a recording mechanism (Shipman et al., 2017). The authors wrote a short movie sequence into the bacterias’ DNA and read it out afterwards, demonstrating the capability of living systems to store complex and human-made data types in DNA.

For a neurogenomic computer, one would implement one of the above systems which translates neural firing into specific nucleotide sequences and then use deep sequencing (Shendure and Ji, 2008; Malone and Oliver, 2011) to read the information offline (Figure 5B). To keep the neural network more intact, one might even harvest single “recorder neurons,” replace them with neural stem cells so that the network can reconstitute its function and extract the computation results by single-cell DNA sequencing. Afterwards, the information could be archived by various simple methods such as freezing (stable over millennia) or transformed into digital electrical patterns or translated back into neural activity. As information in DNA is generally very stable [i.e., thousands of years under the right conditions (Paabo et al., 2004)] and extremely compact [i.e. petabits per mm^3^ (Church et al., 2012)] this system would represent an enormous leap forward in our ability to process and store large amounts of data. As mentioned in paragraph 3, it is also possible to use DNA to directly influence neural network behavior which might be leveraged to acutely change a neuron’s DNA make-up to modify computational rules. As DNA is increasingly easily and cheaply synthesized (Kosuri and Church, 2014), it will be possible to print out custom DNA stretches that change network function in a desired way.

A first step in generating a neurogenomic computer would be to use *in vitro* neural networks and DNA-based logic circuits in each cell or a subset of cells to perform intertwined network and molecular computations, thereby unlocking the combined capacity of these two individually already powerful entities. The molecular computations within neurons could be realized in various ways, three of which will be discussed here.

**Figure 6:**
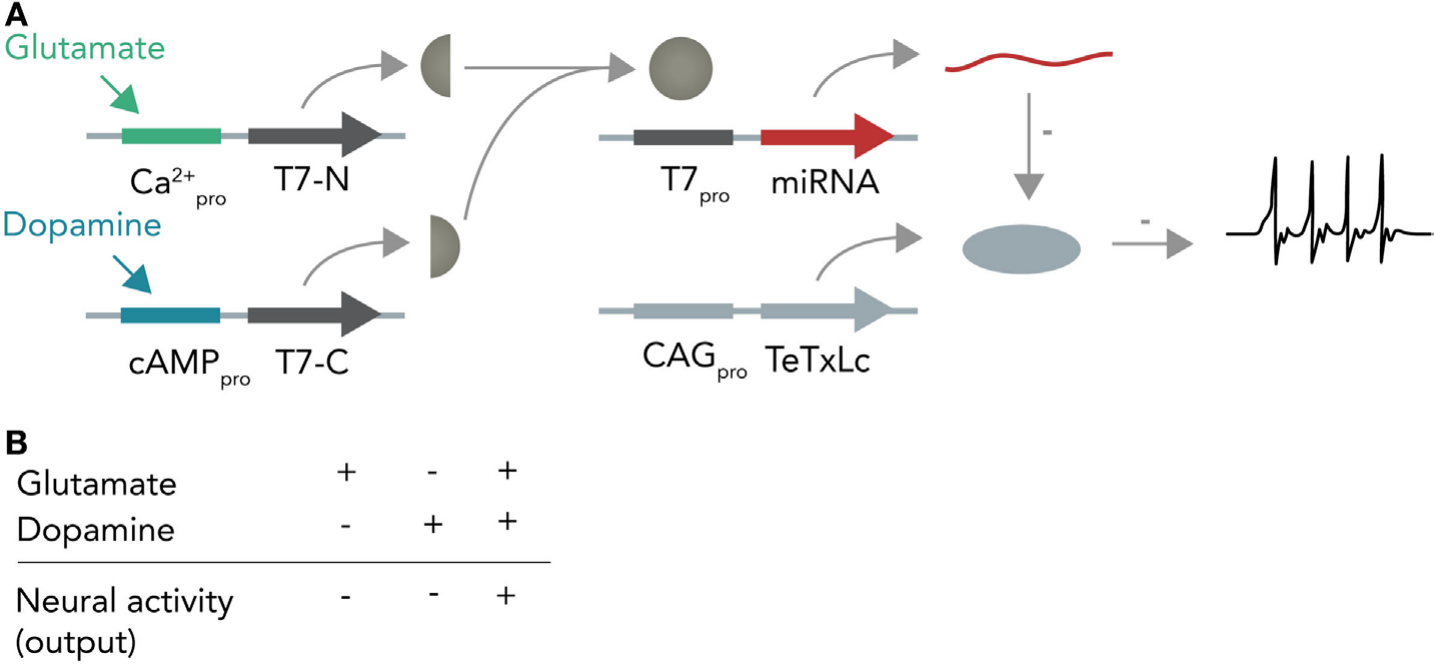
Hypothetical neural activity-dependent DNA-based logic circuit. **(A)** Neurotransmitter-based logical AND-gate. Tetanus toxin light chain (TeTxLc) is constitutively expressed by a constitutively active CAG promoter and silences the neuron under basal conditions. The N-terminal part of the T7 RNA polymerase is placed behind a Ca^2+^-responsive promoter, whereas the C-terminal part is placed behind a cAMP-responsive promoter. When both promoters are activated (i.e. by co-stimulation of the neuron with glutamate and dopamine), both halves are expressed and can form a functional holoenzyme. This holoenzyme now transcribes a micro-RNA against TeTxLc from its specific promoter (T7pro) that leads to degradation of TeTxLc. By degrading TeTxLc the neuron gets activated and can release neurotransmitter onto target neurons. **(B)** Input–output table. Shown is how the different input combinations affect neural output.

In the first instance, one can imagine a DNA-based Boolean logic circuit as commonly implemented in bacterial cells (Khalil and Collins, 2010; Siuti et al., 2013) that is coupled to molecular inputs from neurotransmitter-sensitive cascades and that has neuromodulating molecules as outputs. In the hypothetical example in Figure 6, one calcium responsive promoter element and one cAMP-responsive promoter element [i.e., from input-selective enhancer elements in the c-Fos promoter (Joo et al., 2016) or from different genes such as Npas4 and Arc, both of which react to different input stimuli (Ramamoorthi et al., 2011)] combined with a minimal promoter each drive expression of one half of a split T7 RNA polymerase (Shis and Bennett, 2013). Glutamate will only activate the Ca^2+^-sensitive promoter while dopamine will only activate the cAMP-responsive promoter. The polymerase, if both halves are present, then transcribes a micro-RNA against the constitutively expressed neuronal silencing protein tetanus toxin light-chain (Sweeney et al., 1995) from an orthogonal T7-specific promoter. This system functions as a logical AND-gate in which a neuron is context-dependently activated only if it receives input from both the glutamate and dopamine circuit. As soon as one circuit ceases input, the neuron (within the temporal confinements of protein turnover) will stop participating in the circuit and enter the resting state. Such a system could be used as an oscillator (i.e., if both circuits receive inhibitory input from this neuron) or as a switchable control gate for downstream circuits.

**Figure 7:**
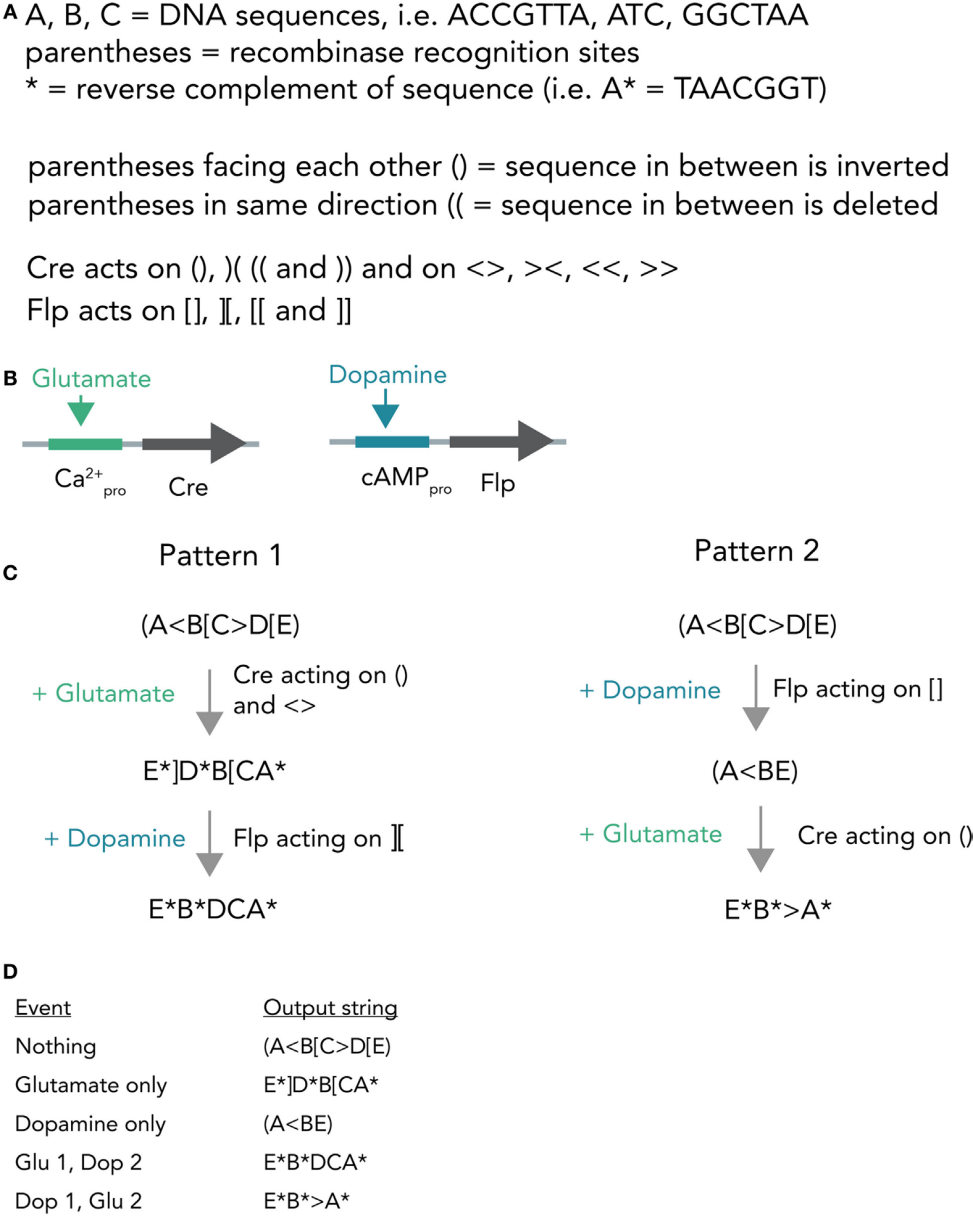
Hypothetical recombinase-dependent neurogenomic computer. **(A)** Notation and rules for the DNA-based computer. Formal notation scheme adapted from (https://www.scottaaronson.com/blog/). **(B)** Expression system. Cre-recombinase is placed behind a Ca^2+^-sensitive promoter and, thus, selectively transcribed by glutamate application. Flp-recombinase is placed behind a cAMP-responsive promoter and thus transcribed after dopamine stimulation. **(C)** Computational process. The neuron starts with a template strand A(<B[C>D[E). If glutamate is applied, this results in transcription of Cre which in turn acts on () and < >. The resulting string reads as E*]D*B[CA* (Pattern 1). In case dopamine is applied afterwards, resulting in transcription of Flp, this sequence is further modified to give E*B*DCA*. Application of dopamine results in (A<BE) and if glutamate is applied afterwards the string is further modified to E*B*>A*. **(D)** Table connecting activity patterns to output strings. The recombination system allows for unambiguous inference of neural activity patterns *post hoc* by sequencing the output strings (i.e., E*B*DCA* clearly means that glutamate was transmitted and then dopamine).

A different hypothetical system makes use of distinct recombinases acting on a constitutively expressed DNA string to produce different unique output strings (Figure 7A). In bacterial cells, such a system was already realized and it was able to record various sequences of outside stimuli into DNA (Roquet et al., 2016). Combining this system with neuron-compatible recombinases, which are able to perform Boolean logic operations in neurons even *in vivo* (Fenno et al., 2014), and expressing them in neuronal networks, could allow the recording of neuronal activity patterns in DNA. In short, Cre and Flp recombinases are placed behind either a Ca^2+^- or cAMP-responsive promoter (Figure 7B). If glutamate is applied, this leads to Ca^2+^ influx and, thus, transcription of Cre. In case dopamine is transmitted, the result is Flp transcription. Both recombinases act on unique target sequences to transform the input string into one of several output strings. Importantly, the sequences of the output strings depend on the exact temporal order in which the neurotransmitters were applied (Figures 7C,D). The DNA string thus holds a memory trace of the different inputs over time. Figure 7D shows how each output string allows inference of the previous activity pattern.

**Figure 8:**
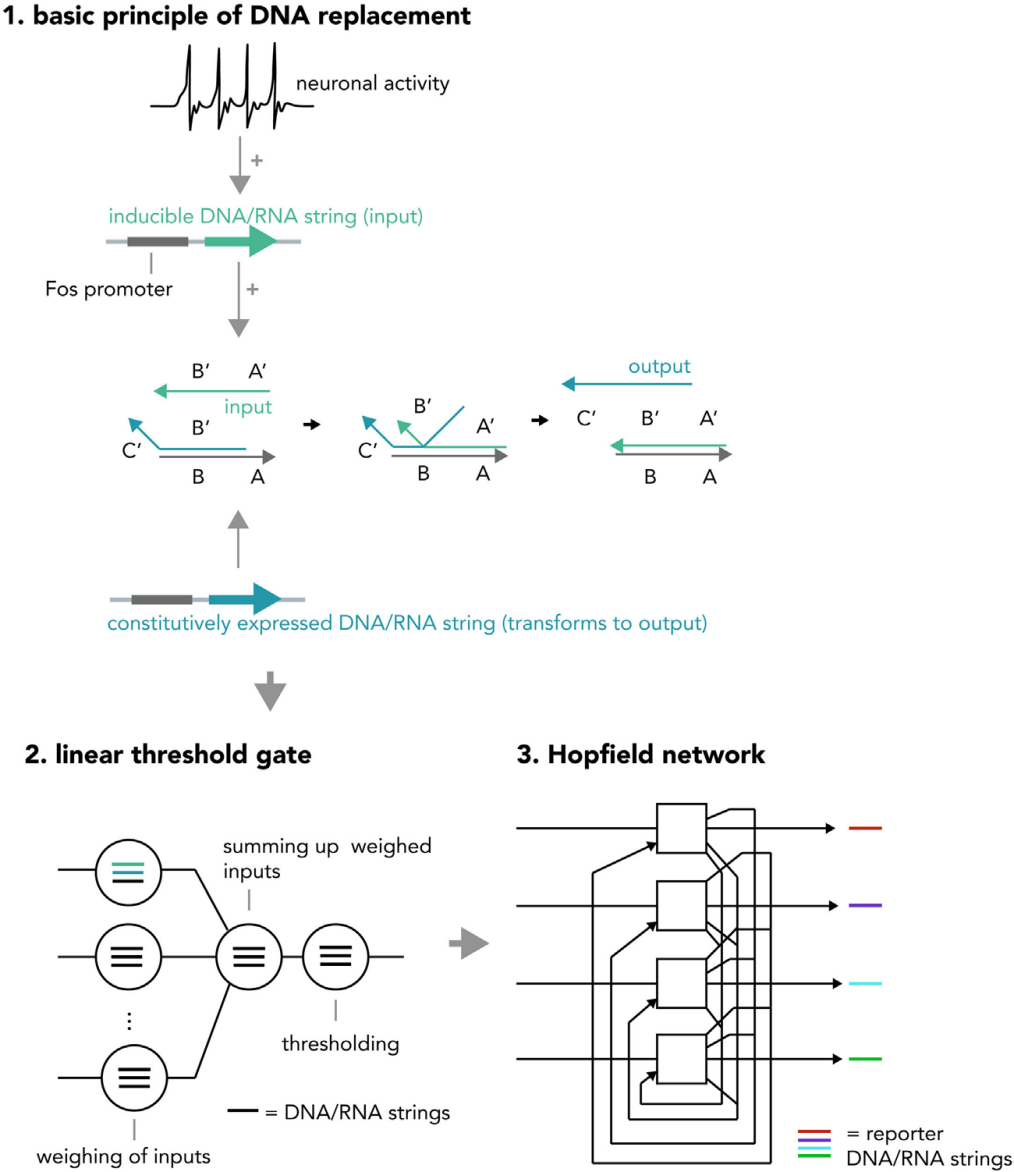
Hypothetical neural activity-dependent strand replacement cascades implementing a “neural network within a neuron” approach. (1) The basic principle of a displacement cascade. A template RNA or DNA strand containing sequences A and B as well as a strand containing a partially complementary sequence B′ (output strand) are constitutively expressed. The output strand binds to the template under basal conditions and cannot exert downstream functions. When the neuron is activated, a new string consisting of sequences A′ and B′ (input string) is transcribed (RNA) or synthesized (DNA) and displaces the output string. The output string can now exert downstream functions (i.e., as an input to another cascade or to be analyzed via PCR). Note that this figure represents a simplified system and in order to implement an actual seesaw-gate motif as done in a previous study (Qian et al., 2011), additional components are required (i.e., fuel and threshold strings). (2) Different displacement reactions (circles) with different functions (weighing of inputs, summing up weighed inputs, thresholding) are combined to create a seesaw gate acting as a linear threshold gate. (3) These seesaw gates can be combined to create a Hopfield network, serving as an associative memory. The state of each “neuron” is reported by an output DNA string (i.e., one that replaces a quencher from a fluorophore-tagged string and thereby causes a fluorescence increase). If expressed inside a neuronal cell, the above DNA system realizes a “neural network within a neuron” approach and could increase the ability to compute information with biological neural networks or implement complex pre-determined plasticity rules (i.e., by creating output strings that incorporate DNA/RNA coding for neuronal activity-related proteins). One could also use these circuits in cellular therapeutic approaches, by implementing them as intelligent sensors that modify neural activity based on pre-determined rules (i.e., induce burst firing if (1) glutamate is received, (2) cortisol is present, and (3) a pharmacological compound is administered).

Another system for multi-layered, parallel information processing would use nucleic acid computation techniques such as strand displacement cascades within each neuron (Figure 8). In short, strand displacement (Qian and Winfree, 2011; Zhang and Seelig, 2011) makes use of the predictable kinetics of Watson–Crick base pairing and realizes inputs and outputs as single stranded DNA or RNA molecules that act via double-stranded intermediates (see Figure 2B). If an input (single strand A) is present at high enough concentrations, it will hybridize with an existing partner in a double-stranded DNA helix and replace and thereby release another single-strand, the output B. This output can be measured directly or be used as an input in a downstream displacement reaction, thereby creating a signaling cascade (Qian et al., 2011; Zhang and Seelig, 2011). A recent report has harnessed these computational abilities to build in test tubes a DNA-based artificial neural network that was able to answer multi-variable questions presented in the form of 4-bit patterns in a reliable fashion (Qian et al., 2011) and another study has demonstrated that predictable nucleic acid replacement cascades can indeed be implemented in mammalian cells (Groves et al., 2016). Combining this technology with *in vitro* neural networks, in which each neuron performs a molecular computation in the form of DNA or RNA strand displacement and then reports output and/or translates it back into network activity might open up new opportunities for information processing in living systems.

Although the prospect of using these technologies is exciting, it might prove difficult to predict the precise functions of this type of circuit, as nowadays even the most simple synthetic biological logic circuits are hard to standardize and predict (Kwok, 2010) [although promising efforts are underway (Nielsen et al., 2016)]. Due to their extremely complex nature, the suitability of neurogenomic modules will most likely have to be determined experimentally with multiplexed approaches (Rogers and Church, 2016) and modified by directed evolution and human-guided learning.

### Opportunities and Challenges of Synthetically Translating Neural Activity into DNA/RNA Changes

#### Opportunities and Advantages

Storing neuronal activity patterns in nucleic acids might have several advantages, some of which have been previously reviewed (Marblestone et al., 2013). First, for basic neurobiology research, it would allow recording a vast amount of neurons in the brain simultaneously with single-cell resolution and reading the results out offline. Recent studies show the possibility of upscaling single-cell genomics and transcriptomics to tens of thousands of cells (Macosko et al., 2015). Neural activity patterns from DNA-barcoded neurons could thus be correlated to transcriptional characteristics (i.e., cell marker expression) and answer long-standing questions about the functions of different cell-types and individual cells in neural circuit physiology and organism behavior. Another advantage might lay in making neural activity recording more cost-efficient and less dependent on biophysical expertise, opening up the possibility of conducting large-scale neural activity recordings for laboratories that would otherwise not be equipped to perform them. As DNA represents an enormously compact and stable storage medium, computations performed by neurons and stored in DNA might circumvent electrical circuits altogether and allow purely biological computers with nucleic acids as the primary storage medium. Furthermore, DNA opens a window to profound control over various aspects of neuronal physiology and thus allows researchers to dictate the rules of neuronal computation. It makes possible a situation where, analogous to modern silicon-based deep learning applications, researchers define critical computational parameters of neurons by writing them in DNA and then let the network find optimal solutions on its own.

#### Challenges and Disadvantages

A major disadvantage of using neural activity–DNA interfaces would be that neurons have limited volumes and can only harbor a certain amount of nucleic acids. For molecular ticker-tapes, a previous report (Marblestone et al., 2013) estimated the capacity for DNA based recording systems with a speed of 1,000 bases/second and 10,000 templates per cell: in a neuron, according to the authors, this would allow 300 s of recording before the transcripts equal the length of the human genome. For RNA with appropriate modifications, this could allow around 2.75 h of recording before an RNA amount equivalent to the physiological one would be reached. Apart from the limited storage capacity, DNA replication and RNA transcription usually pose a major metabolic burden on the cell. They require intense amounts of ATP and other metabolic resources and chain elongation might thus interfere with neuronal physiology and bias experimental results. For recombinase or CRISPR-based recording devices, it is possible that these DNA cutting enzymes would have off-target effects and start modifying the host-cell DNA in unpredictable ways. Another problem for all of the above methods is cellular delivery. How would these multi-component systems be introduced to neurons? Most likely, they would have to be delivered *via* viral vectors or by transgenic means, both of which can be resource-intensive and interfere with the organism’s physiology. A clear bottle-neck for the proposed transcription-dependent systems is the temporal lag through mRNA transcription and possible subsequent protein turnover. This means that calculation results would only be available after some delay, thereby making the system less practical for time-efficient use. It is hence possible that these systems would be more useful in situations where the advantage of parallelizing (i.e. by increasing the number of neurons or networks that could be used for a given task) would outweigh the drawback of low speed. Possible real-life applications could be the encoding of large datasets for archival purposes (i.e. for documentation in blockchains such as Bitcoin) or for tasks where a solution would be valid for a certain, prolonged amount of time (i.e. travel routes through cities).

Another drawback for all of the above systems is that biological processes are still hard to predict and control with high precision. Although efforts to create predictable biological modules are being developed, many synthetic biological circuits and devices fluctuate regarding key functional parameters and might depend on unclear environmental parameters within each laboratory.

## Creating Genome-Customized Neurons to Correct Neural Circuit Pathologies

**Figure 9:**
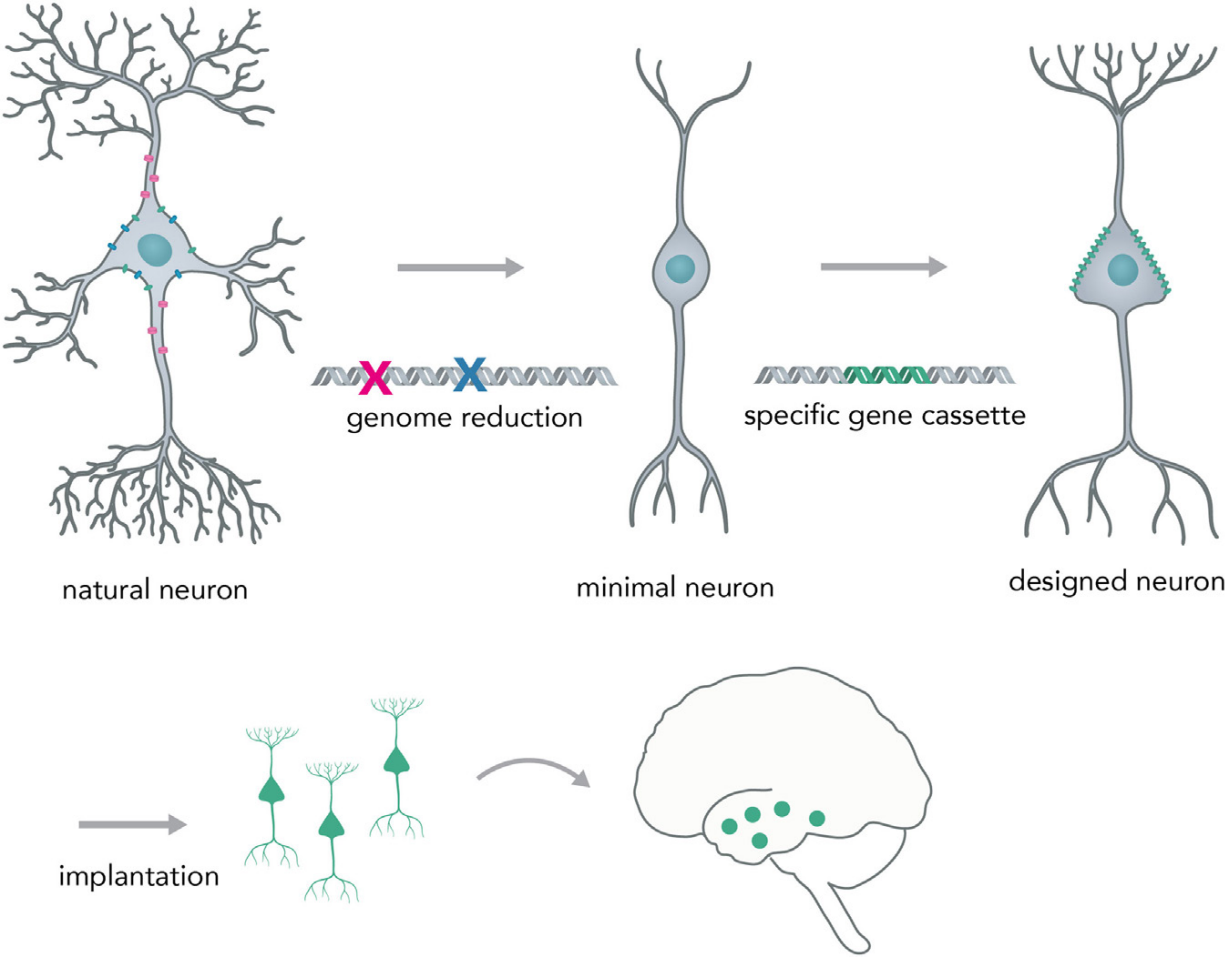
Creating a neural network prosthesis by using the minimal neuron approach. Naturally occurring neurons are highly complex and thereby hard to engineer. In the first step, termed genome reduction, minimal neurons are generated. Genes that are not essential to basal neuronal function are eliminated while others are retained in order to ensure a minimal neuronal phenotype (i.e., maintaining polarity, excitability, and rudimentary synapse formation). In the second step, a gene cassette is introduced to create a neuron with a predictable phenotype (designed neuron). This neuron can then be implanted into the network and will modify activity in a desired fashion (i.e., correct for neural circuit pathologies, such as altered excitation/inhibition balance in epilepsy or autism).

A possible next-generation application in which nucleic acid-controlled neural network behavior might be implemented consists of custom cellular prostheses in the form of modified minimal neurons (Figure 9). Current methods for the *in situ* modification of neural networks have several disadvantages such as lack of targeting specificity (pharmacologicals) or irreversible disturbance of brain physiology (i.e., rAAVs). To circumvent these problems, one could create a minimal neuron template, derived from a patient’s induced pluripotent stem cells, in which all genes that are necessary for the basic neuronal phenotype (i.e., for polar structure with dendrites and axons and rudimentary synapses) are active and other non-essential genes that could lead to unpredictable behavior (i.e., kinase-phosphatase circuits, non-essential ion channels) are deactivated by multiplexed genome deletions [i.e., via Cas9 (Mali et al., 2013) or by synthesizing a reduced genome]. Genomic approaches to create a minimal cell have already been successfully applied to bacteria (Hutchison et al., 2016) and suggest that it is feasible to apply them to mammalian cell types. Based on what the concrete pathophysiology for the individual patient requires, one could synthesize and express a custom-made gene cassette that leads to a neuronal phenotype as required. The cells would be transplanted into the network, as has been successfully done in animal models for human iPSC-derived neurons (Victor et al., 2014), to correct deficiencies in neural computation and diseased phenotype. Prime examples would be pathophysiologies in which the excitatory–inhibitory balance is shifted, such as autism (Nelson and Valakh, 2015) and epilepsy (Fritschy, 2008). Previous studies were able to correct an epileptic phenotype in mice by transplanting inhibitory neuron progenitor cells into the hippocampal subregion (Hunt et al., 2013). By using the minimal neuron template approach, it could be possible to concretely modify any given neuronal circuit in a desired way and thus correct major behavioral pathologies without the drawbacks of modifying existing neurons by viral-mediated protein expression or implantation of physical devices. This approach would heavily benefit from developments in large scale *in situ* genome modifications and cheap synthesis of long DNA segments, as well as from efforts to create human artificial chromosomes. Psychiatrists would be able to prescribe expression units (eu) of genes instead of milligrams of pharmacological compounds (i.e. +1 eu for DNA overexpression of a GABA producing and releasing gene complex in epilepsy) and laboratories within the clinic would be able to print the required DNA segments and transfer them into minimal graft neurons.

## Conclusion

In the course of biological history, evolution has brought together two of nature’s most powerful information processing systems—neural membranes and nucleic acids. Each one is able to compute and store information over many different timescales from sub-second to decades. Recent advances in both genomics and neuroscience have made possible the precise and rapid control and readout in both systems and bring within reach a next generation of combined DNA and neuron-based computational and medical devices. Synthetic biology will immensely benefit from new discoveries in the field of neurogenomics and neurogenomic research will be able to use synthetic biology tools to study and enhance the brain. The possibility of interfacing neural network components and nucleic acids in a controlled and designable fashion will open up new avenues for cost reduction and maximizing efficiency in biotechnology and personalized medicine.

## Author Contributions

The author confirms being the sole contributor of this work and approved it for publication.

## Conflict of Interest Statement

The author declares that the research was conducted in the absence of any commercial or financial relationships that could be construed as a potential conflict of interest.
